# Beyond the visual: using metabarcoding to characterize the hidden reef cryptobiome

**DOI:** 10.1098/rspb.2018.2697

**Published:** 2019-02-13

**Authors:** Susana Carvalho, Eva Aylagas, Rodrigo Villalobos, Yasser Kattan, Michael Berumen, John K. Pearman

**Affiliations:** 1 Red Sea Research Center (RSRC), Biological and Environmental Sciences and Engineering (BESE), King Abdullah University of Science and Technology (KAUST), Thuwal 23955-6900, Saudi Arabia; 2 Environmental Protection Department, Saudi Aramco, Dhahran 31311, Saudi Arabia

**Keywords:** coral reefs, autonomous reef monitoring structures, reef cryptobiome, eDNA biodiversity, cytochrome oxidase I

## Abstract

In an era of coral reef degradation, our knowledge of ecological patterns in reefs is biased towards large conspicuous organisms. The majority of biodiversity, however, inhabits small cryptic spaces within the framework of the reef. To assess this biodiverse community, which we term the ‘reef cryptobiome’, we deployed 87 autonomous reef monitoring structures (ARMS), on 22 reefs across 16 degrees latitude of the Red Sea. Combining ARMS with metabarcoding of the mitochondrial cytochrome oxidase I gene, we reveal a rich community, including the identification of 14 metazoan phyla within 10 416 operational taxonomic units (OTUs). While mobile and sessile subsets were similarly structured along the basin, the main environmental driver was different (particulate organic matter and sea surface temperature, respectively). Distribution patterns of OTUs showed that only 1.5% were present in all reefs, while over half were present in a single reef. On both local and regional scales, the majority of OTUs were rare. The high heterogeneity in community patterns of the reef cryptobiome has implications for reef conservation. Understanding the biodiversity patterns of this critical component of reef functioning will enable a sound knowledge of how coral reefs will respond to future anthropogenic impacts.

## 1. Introduction

The ecological and socio-economic roles of coral reefs in tropical and subtropical regions are unquestionable [1]. Coral reefs are not only among the most diverse ecosystems on Earth but support critical services to humans ranging from shore protection to fisheries, bioactive molecule production, and tourism [2–4]. They comprise an intricate network of habitats and ecological niches that can be highly variable even at the scale of a single reef outcrop. This structural complexity has been highlighted as one of the factors associated with the well-known high levels of biodiversity [5–7]. However, coral reef biodiversity and, therefore, the critical roles it plays in the marine system are being affected by a combination of local and global pressures such as overfishing, eutrophication, sedimentation, and climate change [8–11]. A sound knowledge on patterns of diversity and driving forces are critical for coral reef conservation and human well-being.

The high reef biodiversity is not only reflected in the spectrum of phyla inhabiting coral reefs [12] but also their size ranges (from microbes to megafauna). Most of the research, however, has focused on large biological components that can be assessed during standard scuba-diving reef surveys [13]. Cryptic fauna represented by an array of small-sized organisms associated with the reef framework comprises the majority of the reef biodiversity [14]. They encompass different lifestyles (e.g. sessile, mobile), trophic functions (e.g. suspension feeders [15,16], detritivores [17], predators [18]), and interact in a variety of intra-organism relationships including commensalism, mutualism, and parasitism that are vital to reef functioning [19–21]. However, understanding of the patterns in cryptic diversity within a reef is still very limited. In addition to limited knowledge of the structure and composition of the cryptic diversity within a reef, the distribution across broad spatial scales has received little attention.

The main limiting factors hindering advances in this research area are related to (i) the relatively small sizes of cryptic fauna, (ii) their preferential residence in hidden spaces, and (iii) the lack of standardized approaches to accurately assess this biological component. To investigate the global diversity patterns of reef cryptic communities, a joint effort by the Coral Reef Ecosystem Division (CRED), the Census of Marine Life (CoML), and Census of Coral Reef Ecosystems (CReefs) developed autonomous reef monitoring structures (ARMS). ARMS are long-term artificial substrate units made of a tiered structure of nine stacked polyvinyl chloride (PVC) plates, including a variety of niches (e.g. high light, low light, and various flow regimes). They mimic the structural complexity of a coral reef and attract organisms that are usually hidden in the reef matrix (i.e. cryptic micro-, meio-, and macro-organisms). ARMS enable both the analysis of sessile (species encrusting onto the plates) and mobile organisms (motile specimens colonizing the various niches between the plates) living in these habitats. These artificial structures represent a standardized and non-destructive monitoring tool, which, in combination with the increasingly available and cost-efficient high-throughput sequencing facilities, allows a comparable and fast assessment of cryptic diversity. ARMS have previously been used to describe the eukaryotic cryptic fauna in a variety of locations, but so far these studies have been limited in scale.

Here, we focus on the distribution patterns across a broad spatial scale of what we have named the ‘reef cryptobiome’, which comprises reef organisms inhabiting hidden spaces within the reef matrix. Distribution patterns are often driven by habitat specificity and geographical distribution [22] with other biological components in the marine environment (e.g. plankton [23]), having been shown to be dominated by rare species on both a local and regional scale [23,24]. For example, while species can be abundant in specific locations as a response to their niche requirements, they display limited geographical distribution due to sensitivity to environmental changes [25]. As the rare community has been suggested as a pool of genetic resources [26,27], a better understanding of the capacity of the coral reef cryptic communities to withstand environmental changes will be achieved by revealing the patterns of abundance and rarity. To better understand these distribution patterns across broad spatial scales, we assessed environmental DNA (eDNA) (e.g. DNA from a wide range of sources including whole organisms, cells, diet items, and extracellular DNA), from 87 ARMS deployed on 22 reefs spanning 16**°** of latitude of the Red Sea coast. We characterized the diversity of the reef cryptobiome by targeting a region of the mitochondrial cytochrome oxidase I (COI) gene from the eDNA present in the ARMS. Rather than presenting a simple characterization of the composition of these biological assemblages, we provide insights into the intrinsic patterns of rarity and abundance of the reef cryptobiome. Additionally, the analysis of two different biological traits (sessile and mobile) allowed us to investigate the consistency in the structural and biogeographic patterns of different subsets within the reef diversity.

## 2. Methods

Satellite data were obtained from the NASA Oceancolor website (https://oceancolor.gsfc.nasa.gov/ downloaded 14 February 2017). Monthly averages, at 4 km resolution, were obtained from the MODIS A satellite system for chlorophyll *a*, sea surface temperature (SST), particulate organic matter (POC), and photosynthetic active radiation (PAR). Spatial resolution required that some reefs were assigned to the same grid point.

Triplicate ARMS were deployed on 22 reefs along the eastern coastline of the Red Sea for approximately 2 years in the period between 2013 and 2017 (electronic supplementary material, figure S1 and table S1). The deployment, retrieval, and laboratory processing of the units was undertaken as in Leray & Knowlton [28] (electronic supplementary material, text section SI for details). Briefly, eDNA from the sessile organisms (comprising the homogenized bulk sample scraped from the plates) and the mobile organisms from the two smallest size fractions (106–500 and 500–2000 μm) was extracted and analysed using metabarcoding approaches (see electronic supplementary material, text section SI for more details). Eukaryotic sequences were targeted using a primer set designed to amplify a 313 bp fragment of the COI gene with a versatile primer set [29] using the PCR conditions detailed in Leray & Knowlton [28] (see electronic supplementary material, text section SII for more details). PCR amplicons were prepared for Illumina MiSeq sequencing (electronic supplementary material, text section SII), undertaken at the King Abdullah University of Science and Technology (KAUST) Bioscience Core Laboratory. Sequences used for this study included a subset of those in Pearman *et al.* [30] with new reads deposited at the The National Center for Biotechnology Information (NCBI) short read archive (accession: PRJNA485689). The reads generated on the Illumina MiSeq were processed following Pearman *et al.* [30] and are detailed in electronic supplementary material, text SIII. The dataset was merged as follows: firstly, pooled ARMS consisted of the merging of both traits (sessile and mobile) to give a representation of the total ARMS community. Further, data were subset based on the sample traits (sessile and mobile) of the ARMS community (electronic supplementary material, text SIV).

The composition of the reef crytobiome was assessed in terms of both operational taxonomic units (OTUs) and sequences with the data being processed from the raw OTU table in the R [31] package *phyloseq* [32] (electronic supplementary material, text SV). Differences in the number of OTUs per region were tested using Kruskal–Wallis. Distance similarity plots were constructed using the Jaccard (presence/absence) index and the shortest distance between the coordinates (distHaversine in the package *geosphere* [33]). The degree of uniqueness of the reefs was evaluated by assessing the local contributions to β diversity [34] (electronic supplementary material, text SV). Generalized linear models were used to investigate the effects of the environmental variables on the number of observed OTUs per reef (electronic supplementary material, text SV). Differences in the community patterns (based on Jaccard) within the Red Sea basin between traits were assessed using Mantel test correlations (method: Pearsons). Indicator species for the regions were determined using the R package *indicspecies* [35] (electronic supplementary material, text SV).

To assess distribution patterns, OTUs were classified as locally rare (less than 0.01% in a reef) and locally abundant (greater than 1% in a reef) as defined by Logares *et al*. [36]. Rare and abundant OTUs were determined for the pooled reef community as well as the sessile and mobile components (replicates within reefs combined). To assess differences between the locally rare and abundant subsets, a Kruskal–Wallis test was undertaken on the pairwise comparisons in the Jaccard similarity matrix. Furthermore, the phylogenetic composition was assessed between the two subsets based on the ranks of sequence abundance. To illustrate relationships between reefs for the abundant subset of the community, a dendrogram was produced based on the Jaccard similarity matrix using the *hclust* function in R with the complete method [37]. Visualization was achieved using the *ggplot2* [38], *ggdendro* [39], and *dendextend* [40] packages.

The core community was characterized as OTUs accounting for on average 0.1% relative sequence abundance and 90% of reefs being occupied. The satellite community was determined by an average sequence abundance of less than 0.001% and being only present in a single reef (adapted from [23]).

## 3. Results

### (a) General characterization of the reef cryptobiome

In the present study, we identified 10 416 OTUs; the mobile and sessile traits had 8903 and 4450, respectively. A total of 2937 OTUs were present in both traits. On average, the mobile trait (mean: 684 OTUs; range: 361–1099 OTUs) was almost twice as diverse as the sessile trait (mean: 365 OTUs; range: 142–639 OTUs). At the reef level (triplicate ARMS combined), we observed an average of 1471 OTUs in the reef cryptobiome (electronic supplementary material, figure S1). Along the Red Sea basin, the majority of the OTUs (53%) were only present in one reef with less than 2% of the OTUs (162) being present in all the reefs (electronic supplementary material, figure S2). A similar pattern was observed for the sessile and mobile traits.

**Figure 1.**
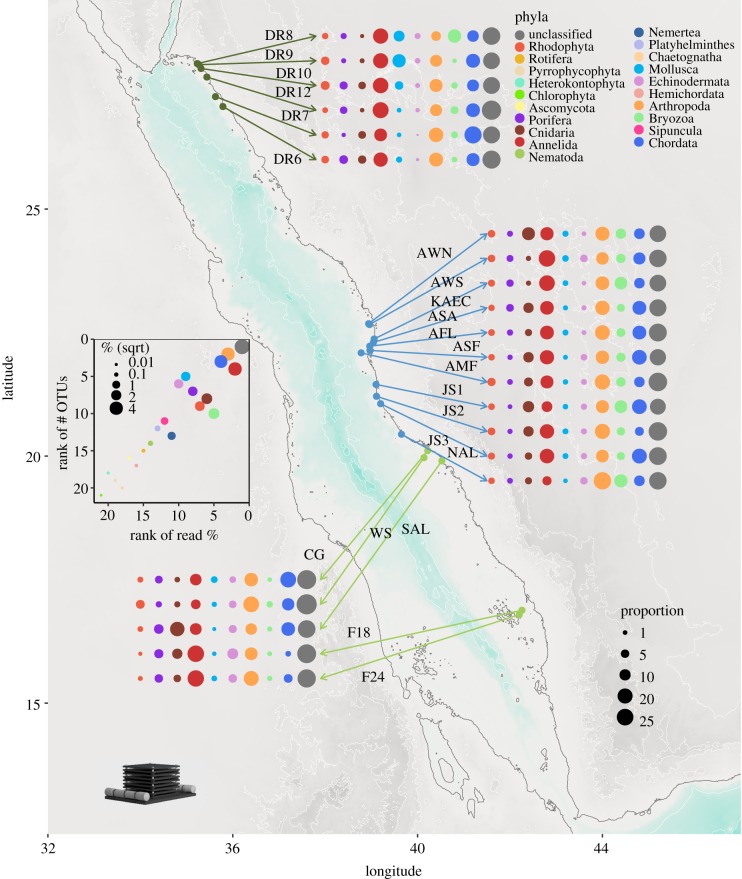
Composition plot of the total ARMS in each reef within the Red Sea. The dots are coloured by phyla and the size is scaled to the proportional abundance within the reef. The inset depicts the relationship in the ranks between the number of OTUs per phyla and the proportional read abundance.

Arthropoda (36% and 16% of OTUs and sequences, respectively) and Annelida (5% and 16% for OTUs and sequences, respectively) dominated the reef cryptobiome community. Mollusca with 3% of the OTUs also showed a relevant contribution to the γ diversity (i.e. the total number of OTUs). Chordata were prevalent in the reef cryptobiome, particularly in terms of the relative proportion of sequences (14%) (figure 1). Other groups with intermediate contributions were Echinodermata, Porifera, Chlorophyta, Bryozoa, and Cnidaria. Approximately 29% of the sequences could not be assigned to any eukaryotic phylum, representing 45% of the OTUs (figure 1; electronic supplementary material, table S2).

With regard to the number of OTUs, the sessile community was dominated by Arthropoda (34%), with these OTUs contributing to 12% of the sequences. Chordata contributed the most to the number of sequences (14%), with Bryozoa accounting for over 10% (electronic supplementary material, table S2). Arthropoda also had the highest contribution in terms of the number of OTUs in the mobile community (38%). Together with Annelida, they also dominated the community with regard to the proportion of sequences (20% Arthropoda; 22% Annelida) (electronic supplementary material, table S2).

### (b) Community composition and structure in space and across traits

The investigation of the similarity patterns of reef communities (Jaccard) showed a significant negative relationship with linear distance (electronic supplementary material, figure S3). Comparative analysis of the community patterns between the sessile and mobile traits showed a significant positive correlation (*p* < 0.001) with a Mantel *R* statistic of 0.55. The assessment of the local contribution to β diversity, based on Jaccard, indicated that the reefs at the extremes of the basin, especially those in the south, showed above average contributions to β diversity (electronic supplementary material, figure S3). This pattern was consistently observed independent of the dataset analysed (i.e. pooled ARMS units; sessile and mobile traits separately). There was a significant difference in the number of OTUs observed in a region for both the pooled ARMS (*χ*^2^ = 22.6; *p* < 0.001) and mobile sets (*χ*^2^ = 25.7; *p* < 0.001) with the southern region having comparatively fewer OTUs. POC contributed the most to explaining the patterns of variance in observed OTUs for the pooled ARMS and the mobile trait and had a negative relationship to OTU number. For the sessile trait, while POC contributed substantially in explaining the observed number of OTUs, SST showed a higher relative contribution (electronic supplementary material, figure S3).

Indicator analysis revealed a total of 14 OTUs characteristic of the northern region; these OTUs were in general negatively correlated to SST and PAR (electronic supplementary material, figure S4). The southern region was characterized by five indicator OTUs, all positively correlated to SST and POC. Arthropoda, Annelida, and Chordata had representative indicators in all regions with Echinodermata represented with one OTU in the northern region.

### (c) Rarity, abundance, and frequency of distribution patterns

**Figure 2.**
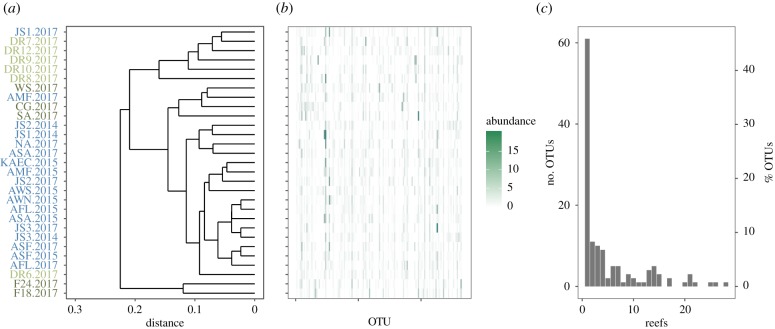
Distribution of locally abundant operational taxonomic units (OTUs) (greater than 1% within a reef). (*a*) Dendrogram indicating the hierarchical clustering (based on the Bray–Curtis dissimilarity) of the abundant OTUs. Reefs are coloured by region (light green, north; blue, central; dark green, south). (*b*) Heat map showing the distribution of the locally abundant OTUs by reef. (*c*) The number of reefs in which an abundant OTU was present.

The reef cryptobiome was characterized by an unbalanced distribution of abundant and rare OTUs. For the pooled dataset, locally abundant OTUs, on average, only accounted for approximately 2% of the diversity at the scale of the reef (table 1), and were generally spatially restricted (no OTUs occurred at all reefs with an abundance greater than 1%). Indeed, of these locally abundant OTUs, 46% were abundant at just a single reef (figure 2*b*,*c*). Hierarchical clustering of the abundant OTUs showed a separation of the most southerly reefs (F18 and F24) with the remaining southern reefs more similar to the central reefs. The northern reefs in the main clustered together (figure 2*a*).

**Table 1.** Minimum, maximum, and mean proportion of abundant and rare OTUs present per site across the different datasets.

	abundant			rare		
	min	max	mean	min	max	mean
pooled	0.8	2.4	1.7	71.4	83.8	78
sessile	3.8	11	6.2	29.2	65.9	51.4
mobile	4	9.2	6.6	37.1	64.7	53.9

On average, 78% of the OTUs of the pooled ARMS were classified in the category ‘locally rare’ (table 1). Similar spatial patterns for the locally rare and abundant OTUs were observed for the sessile and mobile traits (table 1). Ranks of sequence abundance showed that the phylogenetic composition of the sessile community differed between the rare and abundant subsets. For instance, whereas Bryozoa had a comparatively higher rank abundance in the abundant subset, Arthropoda, Mollusca, and Sipuncula were more prevalent in the rare subset. The rare and abundant subsets for the mobile community were more similar. Arthropoda and Annelida are slightly more prevalent in the rare and abundant subsets, respectively (electronic supplementary material, figure S5). There was a significant correlation between the distribution patterns of abundant and rare subsets (pooled ARMS: Mantel *R* = 0.69, *p* < 0.001). However, on average, pairwise comparisons based on Jaccard showed that the abundant subset (average similarity = 0.89) had a significantly higher similarity (*χ*^2^ = 599.27; *p* < 0.001) than the rare subset of OTUs (average similarity = 0.28).

**Figure 3.**
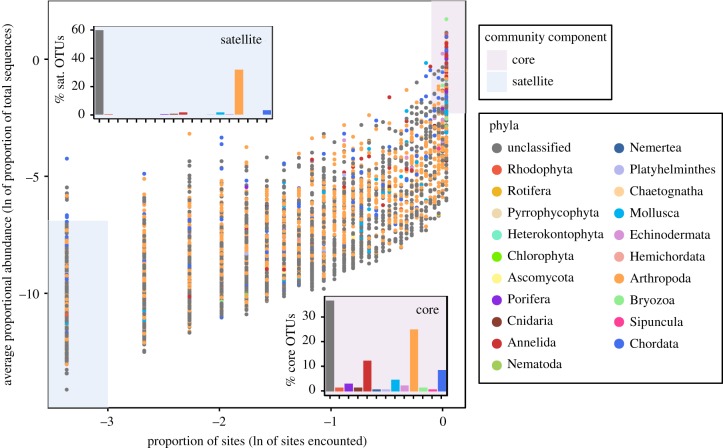
The core–satellite community of ARMS. The average proportion of total sequences against the occupancy of each OTU with the point coloured by taxonomic classification. Insets show the proportion of OTUs classified in each taxa for the core and satellite community.

A significant positive correlation was found between the average relative abundance of an OTU and the persistence of that OTU in the community (Spearman's = 0.84, *p* < 0.001). The core species (i.e. average 0.1% relative sequence abundance and 90% of reefs being occupied) accounted for less than 2% of the γ diversity (128 OTUs), whereas the satellite subset was represented by 52% (5471 OTUs) (figure 3). Similar patterns were observed for both sessile and mobile traits of the reef cryptobiome (electronic supplementary material, table S3). The core Red Sea crytobiome community consisted of 128 OTUs with 36% of the OTUs having no taxonomic classification. Arthropoda (25%), Annelida (13%), and Chordata (9%) contributed substantially to the core community (figure 3). For the satellite community, out of the 5463 OTUs, 32% were assigned to Arthropoda, while 59% were unclassified (figure 3).

## 4. Discussion

In an era where the composition and structure of marine biological communities are changing at increasing rates as a result of anthropogenic activities and climate change, emphasis needs to be placed on understanding patterns across large spatial scales. This fact becomes especially important for groups of organisms that are relatively poorly described and where species could be driven to extinction before their role in the ecosystem is understood [41]. By taking an eDNA approach to assessing the community of ARMS, including both sessile and mobile eukaryotes, we provide a detailed phylogenetic description of what we refer to as the reef cryptobiome. We also demonstrate the potential of this approach to tackle questions at a global scale, namely those related to biological invasions and expansions or contractions in the distributional range of species potentially related to climate change.

### (a) The reef cryptobiome

In the current study, we revealed a total of 20 phyla, of which 14 were metazoans. While multi-phyla studies of the cryptofauna are limited [42], thus making comparisons difficult, the use of eDNA techniques in this study has enabled a broader range of taxa to be revealed. Yet, the main phyla recovered in this dataset are those typically observed in other studies using alternative methods based on the morphological description of cryptofauna during visual census transects [43], or from artificial substrates [44] as well as destructive approaches such as coral heads [21,42], and reef rubble [45]. Arthropoda, Annelida, and Mollusca are the most OTU-rich groups, in agreement with the coral reef biodiversity estimates for mobile cryptofauna [42,46]. Bryozoa, Chordata, Cnidaria, Porifera, and Echinodermata were the other main contributors in terms of either species richness or proportion of reads and have previously been shown to be well represented in crypto-habitats of coral reefs [42,43,47].

The diversity of the reef cryptobiome has likely been underestimated due to a combination of the small size of the organisms as well as the prevalence of nocturnal lifestyles [48], which has led to traditional reef surveys focusing on the more conspicuous corals and fish. Here, the number of OTUs per phyla present in the basin substantially exceeds that of previously described species [49], although this could partially be due to the tendency of metabarcoding studies to inflate the diversity [50]. Furthermore, groups such as Arthropoda, Annelida, and Mollusca have some of the highest estimates for undescribed species [51]. As these groups are prevalent within the reef cryptobiome, it highlights the degree to which this fauna is not fully understood.

Regional differences in the community composition revealed a lower reef cryptobiome diversity in the southern region compared with the central and north. This trend has been previously observed in the Red Sea in plankton communities [52], where nutrients and temperature were shown as drivers of community structure and composition in the Red Sea. Here, we also show that temperature and productivity, indicated by higher POC, are important in the diversity observed in the reef cryptobiome, especially with indicator species in the southern region being positively correlated with POC and those for the north being negatively correlated to temperature.

### (b) Rarity, abundance, and frequency of distribution patterns

Marine communities are in general dominated by a few taxa with most of the diversity resulting from rare species that may change in space and time [23,36].

This trend has been observed from bacterioplankton [23] to soft-sediment macrofauna (0.5–75 mm) [53] and up to fish communities [54]. Here, we confirm this general pattern for the reef cryptobiome as a whole (i.e. sessile and mobile subsets combined) or for each trait separately. Locally abundant taxa were represented by less than 11% of the OTUs within a reef with rare taxa reaching up to 84%. However, a significant and strong correlation in the spatial distribution patterns between the abundant and rare assemblages contrasted to that found for microbial eukaryotes [36]. This suggests that the gradients present within the basin have similar structuring effects on both assemblages despite the differences in their taxonomic composition. The similarity levels in the abundant assemblage were significantly higher than those found for rare species. This is probably due to the fact that the rare community was generally only present in a single sample and thus had a higher spatial turnover.

On a larger spatial scale (e.g. regional), there is a generally acknowledged phenomenon that those species with a higher average abundance are, in general, found more frequently within the community [55,56]. This has led to the core–satellite hypothesis which takes into account the persistence of species within a metapopulation [24]. Here, we reveal a small number of OTUs comprising the core community within the reef cryptobiome of the Red Sea. While there were relatively few core OTUs, they included representatives of 12 phyla with OTUs classified as Arthropoda, Annelida, and Chordata being especially prevalent. This suggests that within the reef cryptobiome, species across a broad range of taxa conform to generalist concepts enabling them to maintain high levels of site occupancy and through positive feedback mechanisms high population sizes [23]. The low proportion of core OTUs indicates a high level of isolation between coral reef communities [57]. On the other hand, satellite species have previously been shown to not be randomly distributed [53] but responded to habitat characteristics. Indeed, a highly specialized species, with a narrow niche preference, will be abundant in a few sites but rare in the majority [26]. The large number of rare species has been proposed to provide a pool of genetic resources that allow functional insurance to changing environmental conditions [26,27]. However, to achieve a better understanding of reef functioning, knowledge of the environmental requirements of these satellite species will be critical.

### (c) The mobile and sessile traits

Marine communities are spatially structured based on (i) dispersal limitation, mortality, and ecological interactions and (ii) environmental variation, with the resulting distribution patterns being affected by species functional traits [58,59]. Even though changes in the spatial structure of communities in response to species traits have been reported before [58], here, we did not find spatial differences between the sessile and mobile communities. Indeed, there was a modest positive correlation in the Jaccard dissimilarities between the sessile and mobile subsets of the cryptobiome. This was rather surprising considering the distinct difference in the taxonomic composition of the traits as well as other life-history differences, such as reproductive methods. While distribution patterns were similar, the environmental drivers differed. SST was more important in describing the α diversity of the sessile assemblage, while POC showed a higher influence on the mobile counterpart. However, both SST and POC were increased in the southern region, where a general decrease in diversity was observed. This indicates that in light of climate change, more research needs to be undertaken to understand the effects of SST on diversity, especially when associated with higher productivity. This highlights the need for an integrated monitoring of reef health encompassing multiple biological components of the reef community (e.g. from bacteria to megafauna) as each component is likely to respond to environmental drivers in different ways [60] and across timescales [61]. Thus, future studies should investigate the responses of taxa across several orders of magnitude of size to fully understand how coral reefs will respond to anthropogenic impacts.

Further differences in the traits were observed in their diversity with distinctly fewer OTUs being present in the sessile community. The lower diversity in the sessile community may be due to the limited space and niches available for sessile organisms in the ARMS (light, shaded with water flow, and shaded without water flow) and the artificial nature of ARMS that may also restrict settlement of some species. The higher diversity for the mobile trait may in part be due to transient occupation of the ARMS unit (e.g. nocturnal animals seeking shelter) from the surrounding mature reef but could also be due to the creation of multiple niches by the sessile community allowing the higher diversity of the mobile trait.

### (d) Limitations of the current approach and future directions

Despite the clear advantages that eDNA metabarcoding approaches show to reveal a great proportion of the biodiversity, molecular approaches are dependent on reference databases for taxonomic assignments, which are currently limited and biased towards common taxa in well-studied regions [62]. The BOLD database used in this study contains high-quality sequences. Yet, the incorporation of sequences in the NCBI database may increase taxonomic coverage, although with a possible loss in sequence quality [63,64]. The limitation of the reference database, along with the possibility of pseudogenes, chimeric sequences, and sequencing errors, results in a substantial number of OTUs not being assigned at the phyla level [63,65], which was also confirmed in the current study (45% of unclassified OTUs; 29% of the sequences). Here, the rdp classifier was used, as it has advantages over assignments based on BLAST, in assigning reads at higher taxonomic ranks [66]. However, the higher proportion of unclassified reads suggests that improvements can still be made. This could be partially achieved by, for example, lowering confidence limits at higher taxonomic levels [66], although the use of other classifiers such as those incorporated in the MIDORI server [67] or using the lowest common ancestor approach in MEGAN [68] as suggested by Macher *et al*.[64] could improve assignments. However, a combination of traditional taxonomy and molecular barcode data is necessary to further resolve basic information about community composition [69]. Indeed, this has been attempted with the Moorea Biocode Project (https://mooreabiocode.org/) but needs to be expanded to other geographical areas as it is essential to catalogue species and understand their niche preferences before they are driven from existence [41]. The use of a single molecular marker can have its limitations and a multiple marker approach can reveal different phylogenetic groups at different taxonomic resolutions and enable a more comprehensive assessment [70–72]. This has already been undertaken on a limited scale for the reef cryptobiome [30], where the taxonomic composition was found to be different between the two markers used, but diversity patterns were similar. Furthermore, caution is required with the use of metabarcoding data for estimations of OTU abundance, due to unequal amplification efficacy across taxa and the detection of different life-history stages (e.g. larvae and adults) [73]. However, studies have shown that the relative abundance of sequences is generally correlated with specimen biomass [74,75]. While correlations are not perfect and can be poor when PCR biases lead to low amplification of various taxa [76], the results can still provide an indication on the structure of the community.

Moving forward, many interesting questions about the reef cryptobiome remain unanswered, such as: (i) the roles of its elements in ecosystem functioning, (ii) what are the unique and common ecological traits of rare-to-abundant taxa, (iii) how its abundance-occurrence patterns relate to the structure of larger benthic and fish communities, (iv) how does the reef cryptobiome change in time, and (v) what are the main drivers of community composition. To achieve these, further investigations could include the targeting of specific functional genes (e.g. nitrogen fixation genes) using qPCR methodologies for the assessment of functional abilities in the coral reef benthic environment. Functional diversity has been proposed as the way forward in regard to the assessment of natural and anthropogenic impacts in the marine environment [77]. Until the assessment of functional roles for the reef cryptobiome follows that of macroecology, questions related to ecosystem functioning or resilience cannot be fully resolved [78]. Furthermore, network analysis across spatial or temporal scales could give a better idea of the relationships between the organisms inhabiting the reef cryptobiome and thus better inform the roles of specific species within the reef. The creation of a time series using ARMS would be valuable to understand how the reef cryptobiome responds to long-term perturbations in the reef environment (e.g. raising water temperatures or increased CO_2_ concentrations).

## 5. Conclusion

The approach followed here confirms the reef cryptobiome as a phylogenetically rich component of the reef system. We identified 14 out of the 30 metazoan phyla [79] that have been associated with coral reefs; however, of those 16 phyla that were not identified in the current dataset, 11 were missing any representative COI sequences in the reference database. This highlights the urgent requirement to collaborate with taxonomists and increase the breadth of the reference databases. We also showed that the mobile component of the reef cryptobiome is almost twice as OTU rich as the sessile. However, and rather surprising considering the contrasting traits and phylogenetic composition, both assemblages show similar spatial structuring patterns, although the environmental variables driving the structuring may differ.

We argue that ARMS combined with metabarcoding processing provides a standardized and non-destructive approach to describing the reef cryptobiome and should be promoted as a way to investigate patterns across variable spatial (local to global) and temporal (from a couple of years to decadal time series) scales. Ultimately, we believe that incorporating the results from the reef cryptobiome into marine monitoring programmes alongside macrobenthos and fish which are typically studied will provide a better understanding of the reef community and enable more informed management decisions to be taken.

## Supplementary Material

Figure S1

## Supplementary Material

Figure S2

## Supplementary Material

Figure S3

## Supplementary Material

Figure S4

## Supplementary Material

Figure S5

## Supplementary Material

Table S1

## Supplementary Material

Table S2

## Supplementary Material

Supplementary Table S3

## Supplementary Material

Supplementary Text

## Data accessibility

Raw sequence reads were deposited in the NCBI short read archive (accession: PRJNA485689).
