# Supplementary figures and images for: Beyond the visual: using metabarcoding to characterize the hidden reef cryptobiome
Source: Proc Biol Sci. 2019 Feb 13;286(1896):20182697. doi: 10.1098/rspb.2018.2697 (PMC6408595; doi:10.1098/rspb.2018.2697)

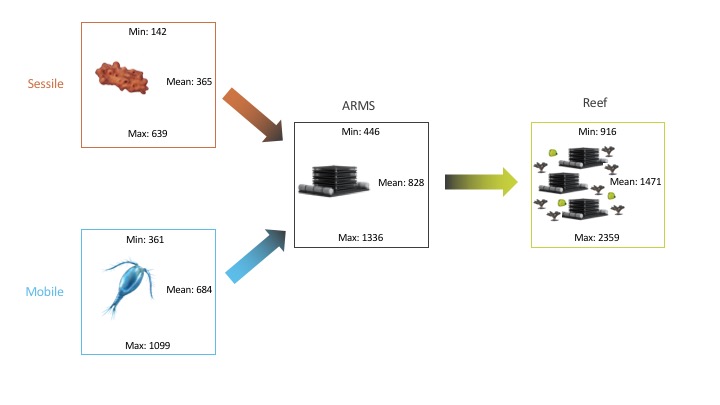

Supplement: Figure S1 [file rspb20182697supp1.jpeg]
