# Supplementary material for: Beyond the visual: using metabarcoding to characterize the hidden reef cryptobiome
Source: Proc Biol Sci. 2019 Feb 13;286(1896):20182697. doi: 10.1098/rspb.2018.2697 (PMC6408595; doi:10.1098/rspb.2018.2697)
Supplement: Supplementary Text [file rspb20182697supp9.docx]

*Carvalho et al.,* **Beyond the visual: using metabarcoding to characterize the hidden reef cryptobiome,** Proceedings of the Royal Society B, doi:10.1098/rspb.2018.2697

ESM File 1

*Figure S1:* ***Alpha diversity at different scales.*** *The minimum, mean and maximum number of OTUs found for the sessile and mobile traits and at the ARMS and reef scales.*

ESM File 2

*Figure S2:* ***Persistence plot indicating the number of ARMS an OTU was present in.*** *A) the pooled dataset; B) the sessile and C) the mobile sub-communities*

ESM File 3

*Figure S3:* ***Patterns of diversity across the basin and environmental drivers****. Similarity (1 – Bray Curtis) against straight-line distance plotted next to the local contribution to beta diversity for each reef and the relative importance of the environmental variables for A) the full ARMS; B) the sessile and C) the mobile traits.*

ESM File 4

*Figure S4:* ***Environmental drivers of indicator OTUs.*** *Indicator OTUs (colored by region: Light Green – North; Blue – Central; Dark Green – South) and the relative importance of the measured environmental variables (darker colors indicate higher relative importance). Significant positive (+) and negative (-) correlations between the abundance of the OTU and the environmental variable are depicted. NS: non significant.*

ESM File 5

*Figure S5:* ***The average sequence abundance of phyla in the rare against abundant fractions*** *for the A) sessile and B) mobile traits.*

ESM File 6

Supplementary Text: Supplementary methodology text.

ESM File 7

Supplementary Table S1: Metadata of the reefs where ARMS units were positioned along the latitudinal gradient of the Saudi Arabian Red Sea

ESM File 8

Table S2: Proportion of reads and OTU’s per phyla

ESM File 9

Supplementary Table S3: Total number of Operational Taxonomic Units (#OTUs) and relative proportion (%) of core, transitional and satellite OTUs across the different datasets
